# Memory recall errors reflect interacting sensory and mnemonic representations

**DOI:** 10.3758/s13423-026-02979-4

**Published:** 2026-08-10

**Authors:** Holly Kular, John T. Serences

**Affiliations:** 1 Department of Psychology, University of California, San Diego, 9500 Gilman Drive, La Jolla, CA 92093-0109 USA; 2 Neurosciences Graduate Program, University of California, San Diego, La Jolla, CA 92093-1090 USA

**Keywords:** Working memory, Neural encoding, Cognitive psychology, Visual perception

## Abstract

Visual working memory (WM) enables the maintenance of information that is no longer present in the environment. Some accounts propose that WM is supported by abstract representations so that new sensory inputs do not interfere with existing memories. Others posit that early sensory representations are sometimes recruited to maintain memory precision, potentially at the cost of interference caused by new inputs. Here we tested these accounts using an orientation recall task to determine whether memory errors reflect interacting representations of sensory and mnemonic information. We found that adding noise to the memoranda and presenting feature-neutral distractors independently increased mean recall errors. However, distractors that shared a feature with remembered stimuli led to systematic attractive biases such that memory errors were pulled toward the orientation of the distractor. The magnitude of this bias was modulated by both stimulus noise and whether the distractor was behaviorally relevant. Our results demonstrate that WM is susceptible to feature-specific interference, suggesting partial overlap between representations of processed and remembered stimuli.

**Supplementary Information:**

The online version contains supplementary material available at https://doi.org/10.3758/s13423-026-02979-4.

## Introduction

Visual working memory (WM) flexibly supports a wide range of cognitive tasks by maintaining information that is no longer present in the environment to guide decision-making and action planning. There are two prominent theoretical frameworks regarding how visual WM representations are maintained in the brain. One perspective argues that WM is supported primarily by neural populations in frontal and parietal cortices that maintain representations that are abstracted away from low-level sensory features (Bettencourt & Xu, 2016; Fuster & Alexander, 1971; Goldman-Rakic, 1995; Mendoza-Halliday et al., 2014; Riley & Constantinidis, 2015; Xu, 2017, 2020, 2024). An alternative, and not mutually exclusive, view suggests that WM also relies on representations that share some commonalities with representations formed during early perceptual processing (D’Esposito, 2007; D’Esposito & Postle, 2015; Harrison & Tong, 2009; Serences, 2016; Serences et al., 2009). That said, frameworks that allow for sensory recruitment and those that do not make distinct predictions about how memory representations interact with new stimulus inputs. If WM relies exclusively on abstract formats in frontal-parietal regions, then ongoing sensory processing should not interfere with stored mnemonic information (Bettencourt & Xu, 2016; Mendoza-Halliday et al., 2014; Xu, 2017, 2020, 2024). However, if perceptual-like representations partially support WM, then representations of continuously visible stimuli and internally maintained representations of remembered stimuli should systematically interact.

Previous fMRI research has shown that mnemonic information is encoded in activation patterns in visual cortex (Christophel et al., 2018; Harrison & Tong, 2009; Rademaker et al., 2019; Serences, 2016; Serences et al., 2009; Sprague et al., 2014, 2016; Sreenivasan et al., 2014). While the structure of mnemonic and sensory representations may not be identical (Kiyonaga & Serences, 2025; Libby & Buschman, 2021), early sensory areas may play an important role when remembering fine details, as neurons in these areas are capable of supporting high-precision representations of relevant information (Harrison & Tong, 2009; Serences, 2016; Serences et al., 2009). Moreover, other studies have shown that distractors can interfere with and attractively bias representations of remembered stimuli, particularly when distractors and the memoranda come from the same feature domain (Dubé et al., 2014; Hallenbeck et al., 2021; Huang & Sekuler, 2010; Lorenc et al., 2018; Mallett et al., 2020; Nemes et al., 2011, 2012; Rademaker et al., 2015; Teng & Kravitz, 2019; Van der Stigchel et al., 2007; Zhang & Lewis-Peacock, 2023). These behavioral effects may arise from interacting, or *entangled,* representations of sensory and mnemonic information (DiCarlo & Cox, 2007; DiCarlo et al., 2012; Pagan et al., 2013). Consistent with this account, Hallenbeck et al. (2021) observed that behavioral reports of a remembered spatial position were attracted to a nearby distractor, as were neural representations of the remembered position in early visual areas V1–V3 as assessed with fMRI.

However, given that distractor resistance is a key feature of WM (Lorenc et al., 2021), some have reasonably argued against sensory recruitment on the basis that repurposing sensory neurons to support mnemonic information could lead to interference between seen and remembered stimuli (Bettencourt & Xu, 2016; Mendoza-Halliday et al., 2014; Xu, 2017, 2020). This is an important concern because we typically move our eyes several times a second and this barrage of new sensory inputs might overwrite mnemonic representations in early visual cortex. Indeed, prior studies using complex objects have reported little effect of irrelevant distractors on memory recall error or on neural representations in early visual cortex, leading to the proposal that WM is supported by more abstract codes in parietal cortex that are reformatted and ‘untangled’ from the feature-specific content of new sensory inputs (Bettencourt & Xu, 2016; Xu, 2024).

Here we test whether sensory and mnemonic representations systematically interact, and the extent to which actively processing the distractors modulates any interactions. In a simple recall task for a remembered orientation, we presented either no distractor or a distractor that could be irrelevant and ignored or that could be task relevant and require active processing and a response. Additionally, we manipulated the sensory uncertainty of the memory sample as a means of parametrically varying memory strength. By using a well-characterized stimulus space, we could make precise predictions about the form of potential interactions between sensory and mnemonic representations (e.g., attractive biases between remembered and seen orientations). Moreover, by varying sensory uncertainty we could evaluate the relationship between memory fidelity and susceptibility to bias and we could establish sensitivity to detect distractor-induced changes in recall error should any exist. For example, prior work demonstrates that changing the amount of coherent information in a stimulus reduces the precision of feature-selective population codes in early visual cortex (Newsome et al., 1989; Parker & Newsome, 1998) and that processing relevant features, such as the orientation of a distractor, increases the gain of neurons in early visual cortex (McAdams & Maunsell, 1999; Moran & Desimone, 1985; Reynolds & Chelazzi, 2004; Reynolds & Heeger, 2009; Treue & Martínez Trujillo, 1999). Thus, manipulating sensory noise and distractor relevance should lead to co-occurring modulations in early visual cortex, affording the opportunity to test for interactions between seen and remembered stimuli. If mnemonic representations are abstracted and protected from distractor interference via untangling, then we predict larger recall errors due to changes in sensory noise but no systematic interactions between sensory noise and distractor relevance. However, if WM and sensory representations rely on partially overlapping mechanisms, then we predict that errors should be systematically biased by the presence of feature-similar distractors, particularly when memory strength is low and distractors are actively processed.

## Experiments 1 and 2

In Experiment 1, we assessed whether increased sensory uncertainty associated with a memory stimulus leads to more susceptibility to interference from distractors presented during the delay period. We collected data from 42 participants (21 women, mean age = 20.6 years), who completed the study for course credit or monetary compensation ($15/hour). Participants in the present study were excluded if they failed to respond within a 3,000-ms window on more than 10% of all trials (and the duration of the response window is consistent with similar WM tasks, Rademaker et al., 2019; Sprague et al., 2016). After excluding eight subjects, all analyses were conducted on the remaining 34.

In Experiment 2, we added a distractor-related task that was irrelevant to the main working memory task to determine if depth of processing of the distractor alters patterns of interference. We collected data from 43 participants (33 women, mean age = 20.0 years) who completed the study for course credit or monetary compensation ($15/hr). As in Experiment 1, participants were excluded if they made no response within the 3,000 ms response window on more than 10% of all trials. After excluding eight subjects, all analyses were conducted on the remaining 35. While we were not able to identify sufficiently similar prior studies to firmly motivate the required sample size, we did find studies that manipulated either stimulus noise or distractor salience/relevance, but not both (with sample sizes varying from ~ 7–21 participants; Clapp et al., 2010; Hallenbeck et al., 2021; Rademaker et al., 2015; Tomić & Bays, 2018). As a result, the sample size for Experiments 1 and 2 was estimated based on these existing studies as well as pilot data we collected in simpler studies, and we aimed to collect data from 40 participants in each experiment (and ended up collecting data from several additional due to asynchronous signups).

### Task

**Fig. 1:**
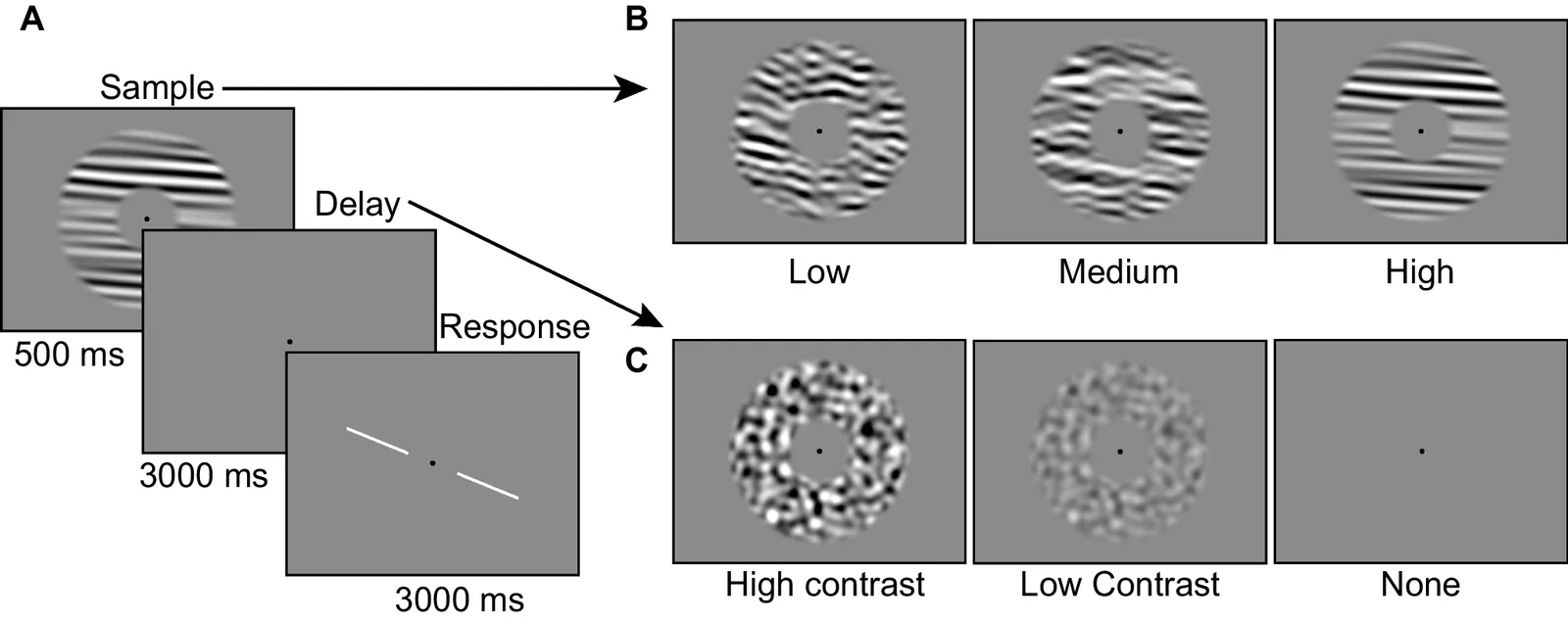
**A** Trial structure for Experiments 1–2. **B** Sample stimulus noise levels. **C** Distractor contrast levels for Experiment 1

Participants encoded the orientation of a sample stimulus presented for 500 ms and retained the information across a 3,000-ms delay period. During the delay, participants saw either a blank screen (no distractor), a 50% Michelson contrast filtered noise distractor, or a 100% Michelson contrast filtered noise distractor (Fig. 1C). After the retention interval, the orientation of the response probe was chosen randomly with respect to the orientation of the sample stimulus on each trial. Subjects rotated the white probe line using four buttons on a standard keyboard to match the remembered orientation of the sample stimulus: Two buttons were used to rotate the line to the left/right in large increments and two buttons to rotate the line to the left/right in small increments. This procedure was adopted to facilitate faster responses.

Experiment 2 used the same task as Experiment 1 with the addition of a distractor task so that the distractor condition was either none, ignore, or process. On the ignore-distractor trials, subjects passively viewed the distractor, as in Experiment 1. On the process-distractor trials, subjects were instructed to press a key in response to a brief decrease in the contrast of the distractor that occurred for 125 ms at a uniformly and pseudo-randomly distributed time between 500 ms and 2,500 ms of the 3,000-ms delay period. On both ignore and process trials, the distractor was always a 65% Michelson contrast noise mask.

In Experiments 1 and 2, each run consisted of 36 trials and there were 10 runs for a total of 360 trials per participant collected in a single 1.5-h session. Stimulus noise, orientation, and distractor type were fully counterbalanced across runs. Participants were instructed to fixate on the central point throughout each trial. The uniform sampling across the entire 180° orientation required high-precision memory and thus discouraged verbal encoding strategies. In addition, we further discouraged verbal encoding strategies with explicit instructions for suppression (“repeat ‘the’ in your head”; Nedergaard et al., 2023).

### Stimuli

The sample stimulus was a phase-reversing orientation, rendered in a circular shape (7° radius), with a circular annulus around a central fixation point cropped out of the stimulus (2° radius annulus and 0.2° fixation; Fig. 1A). The orientation information within the stimulus was parametrically manipulated with a bandpass filter with the width of the filter determining the concentration of orientation information in the stimulus. First, we generated a white-noise image that was 520 × 520 pixels by making random draws from a normal distribution with a mean of 0 and a variance of 1. The image was then transformed into the frequency domain using a fast Fourier transform (implemented in MATLAB, Version 2023a and the “fft2.m” function). We then multiplied the frequency-domain representation with an orientation filter consisting of a circular normal (Von Mises) function centered at the desired orientation, with concentration parameters (k) of 50, 100 and 5,000 for the low, medium, and high noise stimuli, respectively (Fig. 1B). We then applied a spatial frequency bandpass filter from 1 to 4 cycles/pixels, with Gaussian smoothed edges (smoothing *SD* = 0.005 cycles/pixel). After multiplying the frequency domain representation of the white noise stimulus with these filters, we replaced the image's phase information with random values uniformly sampled between − pi to + pi before transforming back into the spatial domain using an inverse Fourier transform (the MATLAB “ifft2.m” function). Finally, we multiplied the image by a scaling factor of the noise image contrast divided by the filtered image contrast in order to account for some of the contrast lost during filtering.

The sample stimulus orientation was first chosen from one of six, 30° orientation bins that uniformly spanned the 180° stimulus space. Sample stimuli were drawn from each of the six bins equally often during a run and once the bin was determined on a trial, the exact orientation was drawn from a uniform distribution across all angles in that bin to ensure that the stimuli evenly tiled orientation space.

The distractors had no bias in orientation information but were filtered to have the same mean spatial frequency composition as the memory sample stimulus following the procedure described above. The distractor was presented as a dynamic image by randomly cycling through a set of 15 independently generated filtered noise stimuli (with replacement) at a rate of 25 Hz.

All stimulus presentation code was written in MATLAB (Version 2023a) using the Psychophysics Toolbox (Version 3.0.19; Brainard, 1997; Kleiner et al., 2007; Pelli, 1997) and all stimulus and analysis code is available online (see *Data availability * section below for links).

### Data analysis

The main dependent measure was the absolute error of the recall responses. We used absolute error as opposed to other measures like the circular standard deviation because absolute error grows with the dispersion of responses and with biases in the mean of the response distribution with respect to the actual stimulus value (where measures such as the circular standard deviation only grow with increased response dispersion). While this feature of absolute error is not critical for Experiments 1 and 2, it is for Experiments 3 and 4 so we adopted this metric across all experiments. All statistics for Experiments 1 and 2 were computed using the R language (Version 2023.06.2) and the *stats* package (Version 3.6.2). We fit log-transformed absolute error using a linear mixed-effects model using the *lme4* package (Version 1.1.35.5) in R. The log transformation was required to satisfy the assumption of constant variance of residuals in linear mixed-effects models (LMM). Finally, we summarized the results using a type 3 ANOVA, using the Satterthwaite method for estimating degrees of freedom from the package *lmerTest* (Version 3.1.3), and calculated effect sizes using partial omega squared and the *effectsize* package (Version 1.0.2). The LMM included fixed effects of stimulus noise, distractor condition, and their interaction. To determine the appropriate random-effects structure, we compared models with progressively more complex subject-level effects using likelihood ratio tests and information criteria. The model including random intercepts for participant and subject-specific condition effects (stimulus noise and distractor condition) provided the best balance of fit and parsimony and was selected for inference. Due to the preregistration of Bayesian ANOVAs for the subsequent experiments, we also report a Bayesian ANOVA of recall error for Experiments 1 and 2 in the Supplementary Materials (Section I).

In order to increase power to detect a potential interaction between stimulus noise and distractors, we repeated the LMM analysis with data combined across Experiments 1 and 2 after excluding trials where there was no condition overlap (i.e., we excluded the low contrast distractor trials from Experiment 1 and the processed distractor trials from Experiment 2, leaving combined data across the no-distractor condition and the high-contrast distractor condition in E1 and the high-contrast ignore distractor condition from E2). The LMM for the combined experiment matched the formula for the individual experiments with the addition of a fixed effect for experiment number. The combined experiment factors remained the same for stimulus noise and were reduced to two levels for the distractor conditions (no distractor, distractor ignored).

### Results

**Fig. 2:**
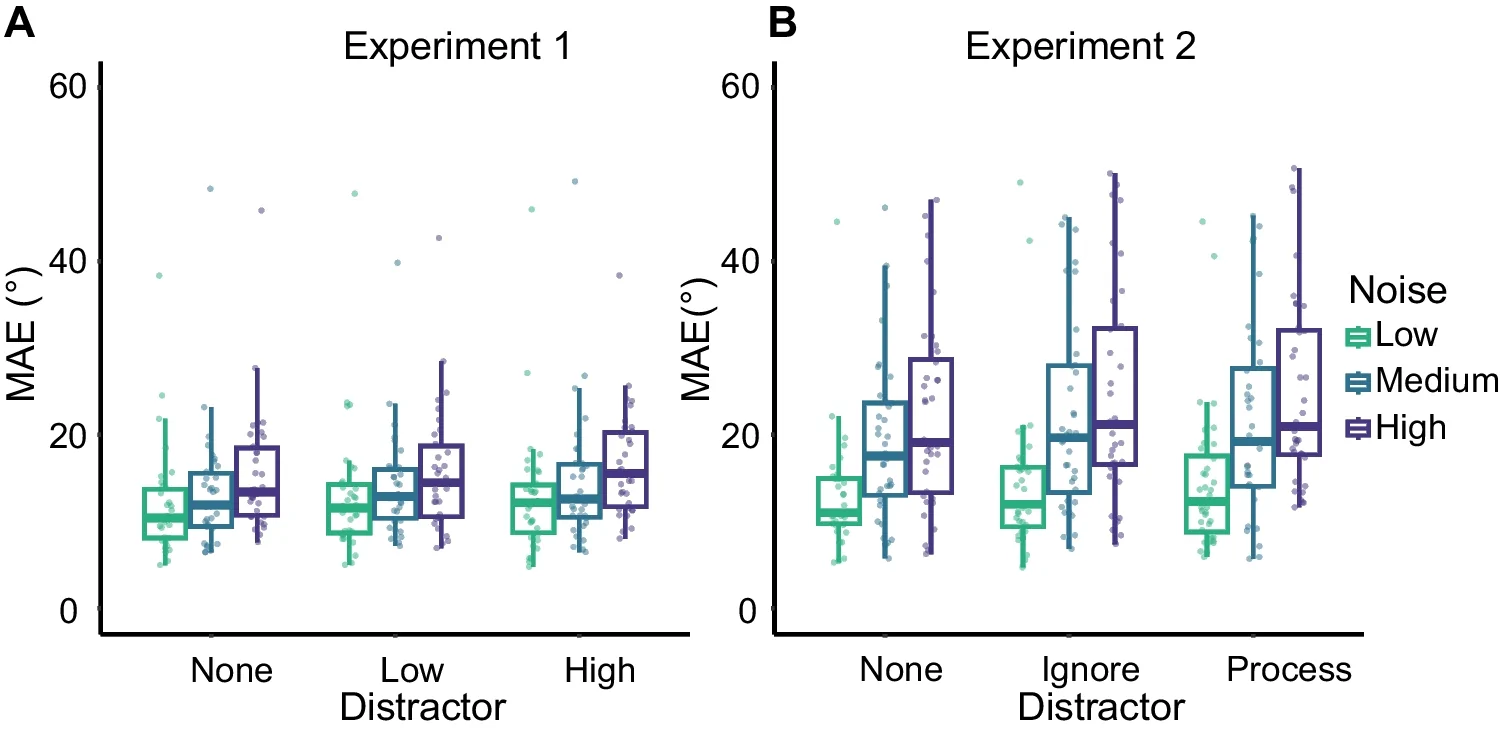
**A–B** Boxplots of response errors (MAE) for each distractor condition (*x*-axis) and sample stimulus noise condition (color). Plots are in the style of Tukey, boxes display interquartile range, and whiskers are 1.5 × quartiles. Individual dots represent individual participant errors. (Color figure online)

Working memory errors were measured using the absolute error of the reported orientation compared with the actual orientation of the memory sample (Fig. 2A). In Experiment 1, the LMM revealed larger errors with higher stimulus noise, *F*(2, 65.5) = 27.6, *p* < 0.01, ω_p_^2^ = 0.44, but no significant main effect of distractor condition, *F*(2, 66.3) = 2.00, *p* =.14, ω_p_^2^ = 0.03. There was no significant interaction between stimulus noise and distractor condition, consistent with additive effects of stimulus noise and distractor condition, *F*(4, 11,732.7) = 0.80, *p* = 0.50, ω_p_^2^ = 0.00.

In Experiment 2, there was a significant main effect of stimulus noise on response errors, *F*(2, 73.3) = 50.5, *p* < 0.01, ω_p_^2^ = 0.56, and a significant main effect of distractor condition, *F*(2, 73.8) = 10.2, *p* < 0.01, ω_p_^2^ = 0.19 (Fig. 2B). Finally, there was no significant interaction between stimulus noise and distractor condition, suggesting that both factors had largely independent effects on response errors, *F*(4,12,480.7) = 1.02, *p* = 0.40, ω_p_^2^ = 5.34 × 10^–6^.

When analyzing data combined across Experiment 1 and 2 (Supplementary Materials: Fig. S6), there was a significant main effect of stimulus noise, *F*(2, 143) = 56.2, *p* < 0.01, ω_p_^2^ = 0.43, distractor condition, *F*(1, 69.3) = 10.9, *p* < 0.01, ω_p_^2^ = 0.12, and experiment number (with larger errors in Experiment 2), *F*(1, 68.1) = 4.95, *p* <.03, ω_p_^2^ = 0.05. As in Experiment 1 and 2 when analyzed separately, the interaction between stimulus noise and distractor condition was not significant, *F*(2, 17,290.7) = 0.69, *p* = 0.50, ω_p_^2^ = 0.00.

## Experiments 3 and 4

Experiments 1 and 2 established that the presence of a stimulus noise led to higher error rates overall, as did the presence of a feature-neutral distractor in Experiment 2. However, there was not an interaction between distractor salience/relevance and stimulus noise. The lack of an interaction is consistent with models suggesting that mnemonic and sensory representations are ‘untangled’, perhaps via reformatting of the remembered information for storage in higher-order cortical areas (Bettencourt & Xu, 2016; Xu, 2024). However, systematic interactions between memory and sensory stimuli could still exist, such as mean-reverting attractive or repulsive biases, without giving rise to an interaction between stimulus noise and distractor salience/relevance on absolute error. Indeed, many prior studies were not designed to assess such feature-selective interactions as they either used different categories of objects as samples and distractors (e.g., Xu, 2024), or, as in our Experiments 1 and 2, used a distractor that did not contain any coherent orientation information. To provide a more direct and sensitive measure of potential interactions, Experiment 3 assessed whether sensory uncertainty interacts with noise distractors that are both behaviorally relevant and from the same feature domain as the remembered stimulus. Experiment 4 was a conceptual replication of Experiment 3, and both were preregistered (see *Availability of data and materials & code availability* section below for links).

Experiment 3 had 24 participants (15 women, mean age = 22.1 years) who each completed 576 trials across two testing sessions totaling ~ 3 h. Experiment 4 had 12 participants (seven women, mean age = 23.4 years) who each completed 1,600 trials in three sessions totaling ~ 5 h. We initially did not conduct a parametric power analysis prior to preregistering Experiment 4 because our planned analyses were non-parametric. For Experiment 4, we initially estimated that with greater than double the number of trials per subject used in Experiment 3, we would be able to detect effects the size of Experiment 3’s with half the participants and so we preregistered 12 subjects. However, after conducting a post hoc parametric power analysis using data from Experiment 3, we determined that the goal sample size should have been 17 participants. In the main text we report results from Experiment 4 with 17 participants (11 women, mean age = 26.2 years). Results from the initial sample of 12 participants are consistent with those presented here (Supplementary Materials: Fig. S5).

### Task

Experiments 3 and 4 were similar to Experiments 1 and 2 but with an oriented distractor and a modified task to perform on the distractor. Experiment 3 had no-distractor, ignore-distractor, and process-distractor conditions that were precued at the start of each trial by changing the color of the fixation point to one of three colors (precue presented for 1,000 ms, and colors counterbalanced across participants). On process-distractor trials, the distractor task required a change discrimination where participants were asked to report whether the distractor rotated clockwise or counterclockwise for using the arrow keys on the keyboard. Since we had a well-established distractor effect in the previous experiments, we dropped the no-distractor condition for Experiment 4. For Experiment 3–4, to ensure task comprehension, participants were required to correctly describe the instructions at the beginning of each data collection session, and they were given feedback during a monitored practice block of trials on the first day of data collection. In addition, we discouraged verbal encoding strategies in both experiments with explicit instructions for suppression (“repeat the cue color in your head”).

### Stimuli

**Fig. 3:**
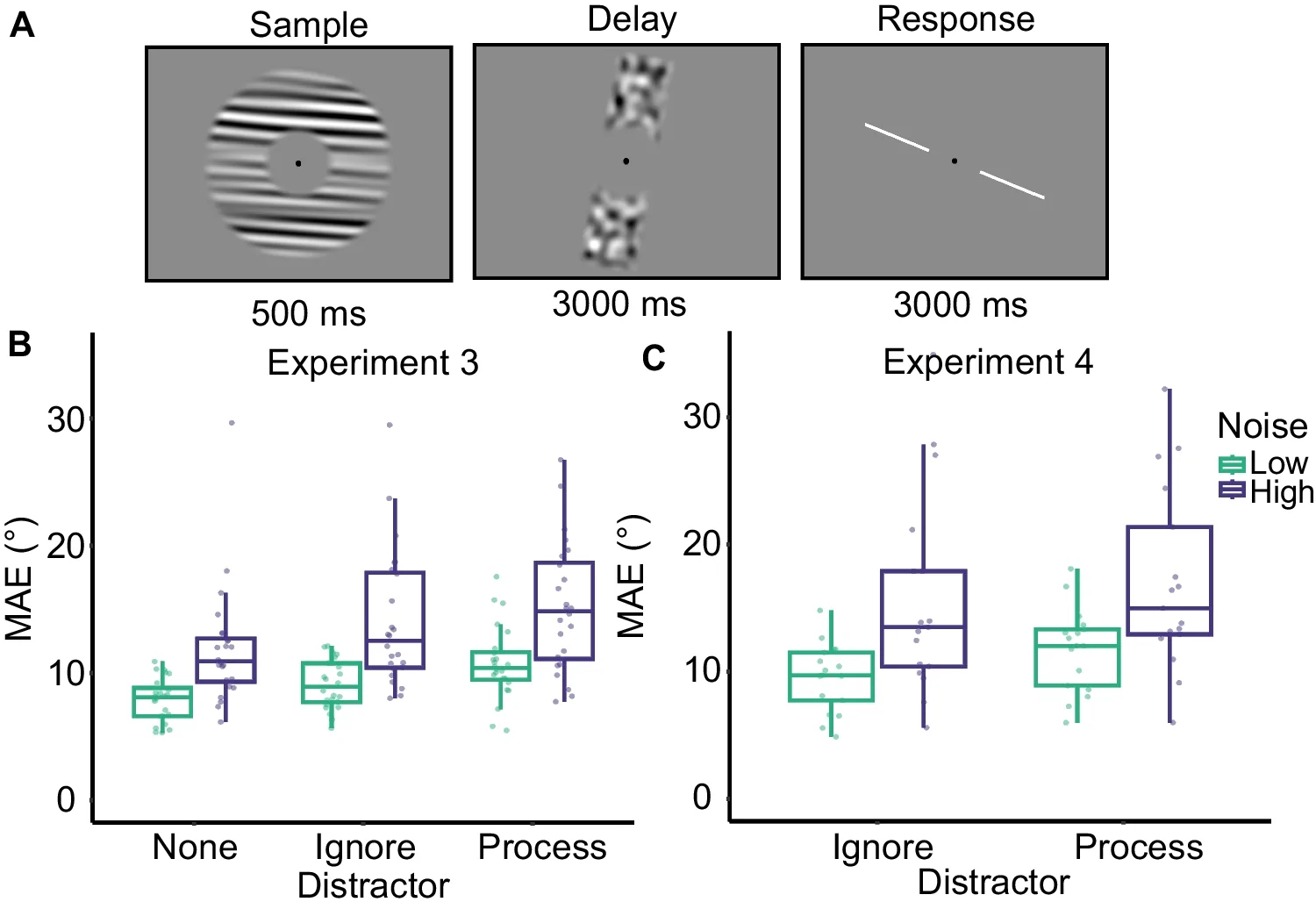
**A** Task structure for Experiments 3–4. Boxplots of response errors for Experiment 3 (**B**) and 4 (**C**). Plots are in the style of Tukey, boxes display interquartile range, and whiskers are 1.5 × quartiles. Individual dots represent individual participant errors. (Color figure online)

The sample stimuli were generated using the same methods described for Experiments 1 and 2. The distractor was an oriented rectangle that was 3.5° wide and 14° long that contained the same dynamic filtered white noise as described in the previous experiments and was rendered at 50% Michelson contrast (Fig. 3A). The sample orientation and the distractor orientation were independently selected from one of six, 30° orientation bins that uniformly spanned the 180° stimulus space (and counterbalancing across runs ensured that sample and distractor orientations were statistically independent across the entire experiment). On each trial the distractor orientation briefly (125 ms) shifted either clockwise or counterclockwise. The size of the shift was initialized at 10° on the first run and was decreased by 1° down to a floor of 2° if distractor task accuracy was greater than 79% (and was increased by 1° if distractor task accuracy was less than 71%, up to a ceiling of 20°). This yielded a mean accuracy = 90.8% ± 7.96%.

The stimuli in Experiment 4 were the same as in Experiment 3, but we sampled the distractor orientation in four 30° wide bins centered at 0°, − 45°, + 45°, and 90° away from the orientation of the sample stimulus to increase the number of trials at these key stimulus-distractor offsets.

### Preregistered exclusion criteria

Following a similar logic to Experiments 1 and 2, participants were excluded if they failed to respond to the main orientation report task on more than 10% of all trials or if they failed to respond to the distractor task on more than 10% of all trials where a response to the distractor was required. Participants were also excluded if they responded during the delay on more than 10% of the trials where there was no distractor task, as this would indicate noncompliance with task instructions. These exclusion criteria were strict but were adopted to ensure that subjects were on task and because we needed enough trials to densely sample all possible combinations of sample/distractor orientation offsets.

After running Experiments 1 and 2 and having to exclude 8 subjects from each, we preregistered that we would continue data collection in Experiment 3 until the preregistered target sample size of 24 participants was reached (with nine participants replaced for failure to respond correctly to the distractor task and one participant replaced for failure to respond to the main task). Similarly, in Experiment 4 data collection continued until the preregistered target sample size of 12 participants was reached (with three participants replaced for failure to respond correctly to the distractor task). After the updated sample size was estimated using Experiment 3’s data, Experiment 4 data collection continued for the additional five participants needed to reach the new goal sample size (with four participants replaced for failure to respond correctly to the distractor task).

### Data analysis

We preregistered that we would perform Bayesian ANOVAs and pairwise comparisons to analyze the root mean squared error (RMSE) of the responses. Following peer-review, response error magnitudes as quantified by absolute error were analyzed using the same linear mixed-effects model approach used in Experiments 1 and 2. Results of the preregistered Bayesian ANOVA and pairwise comparisons were consistent with the unplanned LMM (see Supplementary Materials: Section I and Tables S1–S4). Additionally, we conducted a sensitivity analysis for all experiments to determine the minimum detectable effect size for an interaction (see Supplementary Materials: Section IV).

In order to increase power to detect a potential interaction between stimulus noise and distractors, we repeated the LMM analysis with the combined Experiment 3 and 4 data excluding trials where there was no condition overlap (i.e., the no distractor trials from Experiment 3). The LMM formula was the same as the one used for the individual experiment analysis with the addition of the fixed effect of experiment number.

### Experiment 3: Fitting a Gaussian to folded response errors to measure bias

Because we used a parametrically varying stimulus space and the memory sample and the distractor shared a common feature (orientation), we evaluated whether memory response errors were systematically biased by the distractor orientation using the following procedure:

- **Step 1: Define the memory-distractor orientation offset**. For each trial, we computed the circular difference between the distractor orientation (${\theta}_{D}$) and the sample stimulus orientation (${\theta}_{S}$), ($\Delta \theta ={\theta}_{D}-{\theta}_{S}$).
- **Step 2: Preprocess response errors**. We first excluded trials with no response. We then attenuated cardinal biases by fitting a 4th degree polynomial to the response errors as a function of sample orientation and we used the residuals from that fit for further analysis (van Bergen & Jehee, 2019).
- **Step 3: Compute bias in a moving window**. We estimated bias—defined as the circular mean of the response error distribution—across values of $\Delta \theta$ using a 30° moving window (e.g., a window centered at 0° included all trials with $\Delta \theta$ in the range [− 15, 15°]).
- **Step 4: Folding the bias function**. To increase power, we multiplied each trial’s error by the sign of $\Delta \theta$ and then folded the data around the 0° point before averaging.
- **Step 5: Fit a Gaussian**. We fit the resulting bias function with a Gaussian parameterized with an amplitude a, width w, center of the peak $b=45^\circ$, and *x*’s consisting of the range of $\Delta \theta$.
  1 $$y={a}{e}^{\frac{-{\left(x-b\right)}^{2}}{{2}{w}^{2}}}$$

The two free parameters (a andw) were optimized by minimizing RMSE between the model and the data, first via a grid search and then refined using the Nelder-Mead algorithm (Python 3.12.9, Scipy optimize 1.15.2) with up to 1 × 10^8^ iterations, with bounds of (a[− 5, 8], w[20, 35]).

- **Step 6: Bootstrapping to assess changes in bias**. We fit the Gaussian described in Step 5 using a bootstrap resampling method. First, we generated a sample of pseudo subjects by iteratively taking the average of a random sample of 24 subjects selected with replacement. Next, we calculated a repeated measures ANOVA on this sample of pseudo subjects. We repeated this 5,000 times to construct a distribution of *F* statistics. Note that we used a repeated-measures ANOVA, as opposed to a LMM, because evaluating bias across the distribution of $\Delta \theta$ requires aggregating trials within a subject.
- **Step 7: Permutation testing**. In order to create a null distribution of F statistics to compare to the bootstrapped distribution generated in Step 6, we scrambled the stimulus noise and distractor conditions 5,000 times based on the statistical comparison of interest. To test the main effect of processing the distractor, the null distribution was constructed with just the distractor condition labels shuffled before running each ANOVA. To test the main effect of stimulus noise, the null distribution was constructed with just the stimulus noise labels shuffled. To test the interaction between the stimulus noise and distractor processing, the null distribution was constructed with all labels randomly shuffled.

### Experiment 4: Response error bias estimation

Because we sampled distractor orientations centered on four distractor-sample ($\Delta \theta$) bins (0°, − 45°, + 45°, and 90°), we do not fit a continuous Gaussian function as we did for Experiment 3. Instead, we used the procedures described below.

**Step 1: Fold the error distribution.** First we sorted the response errors by the four distractor-sample ($\Delta \theta$) bins (0°, − 45°, + 45°, and 90°). Next, we multiplied the errors in the − 45°-centered bin by − 1 and averaged them with the + 45°-centered bin. Then we averaged the errors in the 0° and 90° centered bins. This resulted in two $\Delta \theta$ bins – oblique (− 45° and + 45°) and orthogonal (0°and 90°).

**Step 2: Estimate bias amplitude**. Given that we expected attractive biases to be expressed in the oblique bin, we subtracted the mean error in the orthogonal bin from the mean error in the oblique bin as our estimate of response bias amplitude.

**Step 3: Bootstrap resampling and permutation test.** We tested significance using the same bootstrap resampling and permutation test procedures described for Experiment 3 (resample subjects with replacement 5,000 times, perform ANOVAs to generate a distribution of *F* values, compare this distribution of *F* values to a distribution generated with appropriately randomized labels).

### Results of Experiments 3 and 4

#### Mean memory error

As shown in Fig. 3B and C (Supplementary Materials: Fig. S7), working memory errors were larger with more stimulus noise—Fig. 3B, Experiment 3), *F*(1, 23.0) = 25.5, *p* < 0.01, ω_p_^2^ = 0.49; Fig. 3C, Experiment 4: *F*(1, 16) = 13.4, *p* < 0.01, ω_p_^2^ = 0.41; Experiment 3 + 4: *F*(1, 41) = 36.5, *p* < 0.01, ω_p_^2^ = 0.45, and there errors increased with distractor presence/relevance—Experiment 3: *F*(2, 45.8) = 34.6, *p* <.01, ω_p_^2^ = 0.58; Experiment 4: *F*(1, 16) = 17.5, *p* < 0.01, ω_p_^2^ = 0.48; Experiment 3 + 4: *F*(1, 43) = 35.2, *p* < 0.01, ω_p_^2^ = 0.43. Consistent with Experiments 1 and 2, there was no significant interaction between stimulus noise and distractor condition in Experiment 3, *F*(2, 13,509.5) = 0.51, *p* = 0.60, ω_p_^2^ = 0.00. In Experiment 4 and in the combined analysis of Experiments 3 and 4, the interaction reached significance but the effect size was negligible, Experiment 4: *F*(1, 26,904) = 7.65, *p* < 0.05, ω_p_^2^ = 2.47 × 10^–4^; Experiment 3 + 4: *F*(1, 35,906) = 8.19, *p* < 0.01, ω_p_^2^ = 2.00 × 10^–4^.

#### Biases in memory error

**Fig. 4:**
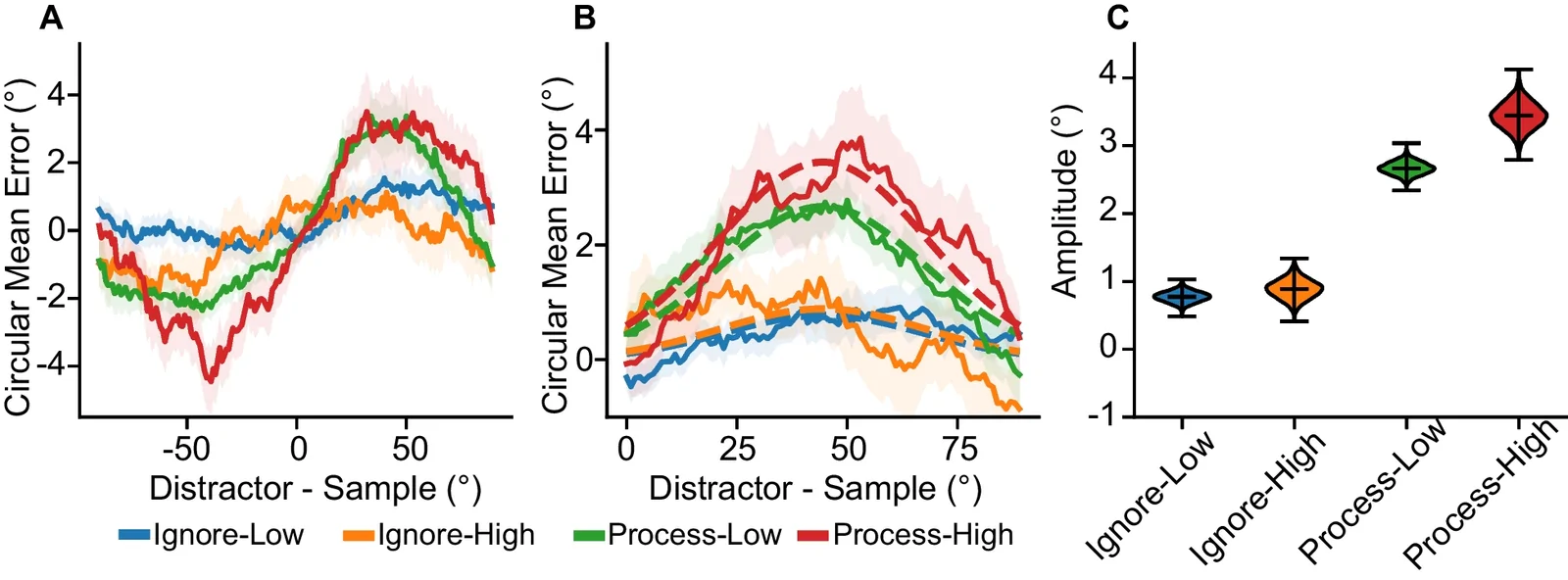
**A** Moving average distractor-induced bias. Shaded region represents *SEM*. **B** Folded distractor-induced bias (solid lines) with fitted Gaussians (dashed lines). **C** Amplitudes of gaussian fits; center bar represents mean and outer bars represent minimum and maximum. (Color figure online)

In Experiment 3, across all conditions, behavioral errors showed an attractive bias towards the distractor orientation (Fig. 4A–B). The magnitude of the bias was larger with higher stimulus noise (permutation test: 95% CI [0.26, 0.63], *p* < 0.001) and when the distractor stimulus was processed (permutation test: 95% CI [1.98, 2.47], *p* < 0.001). Finally, distractor-relevance had a numerically larger impact on response bias for high-noise compared to low-noise stimuli, but the interaction did not reach the traditional 0.05 significance level (permutation test: 95% CI [0.18, 1.17], *p* = 0.06).

**Fig. 5:**
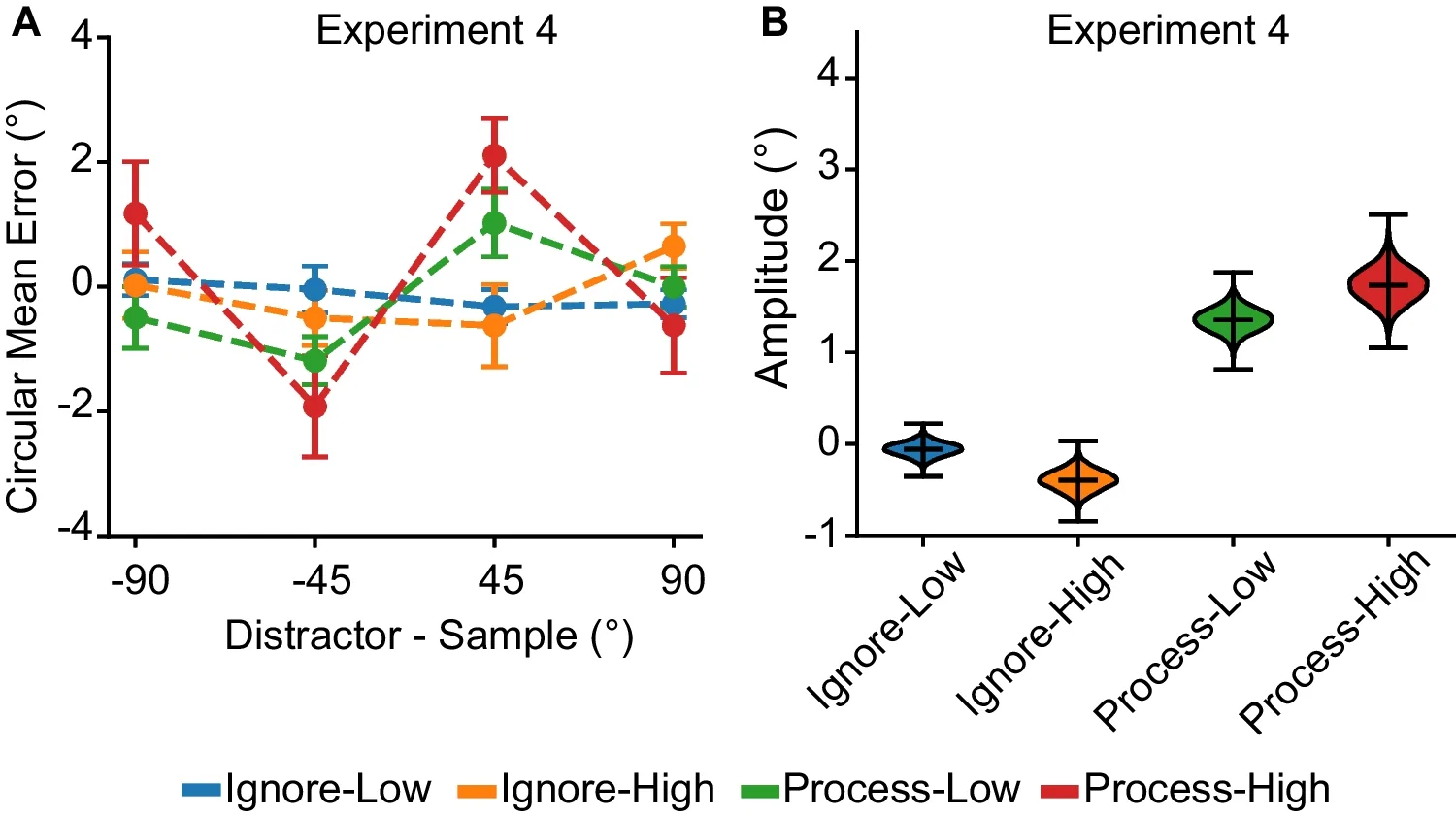
**A** Distractor-induced bias. Error bars are *SEM.* **B** Estimated amplitudes for pseudo-subject samples; center bar is mean and outer bars are minimum and maximum. (Color figure online)

Experiment 4, which used four discrete sample/distractor offsets as opposed to continuous sampling, also reveals systematic biases in memory errors (Fig. 5A–B). The magnitude of the attractive bias between the sample and the distractor was larger when the distractor stimulus was attended (permutation test 95% CI [1.49, 1.95], *p* < 0.001) but there was no main effect of stimulus noise (permutation test 95% CI [− 0.18, 0.42], *p* = 0.36). Consistent with the numerical pattern in Experiment 3, there was a significant interaction such that distractor-relevance had a larger impact on response bias for high-noise compared to low-noise stimuli (permutation test 95% CI [0.39, 0.96], *p* = 0.01).

In order to assess whether the attractive bias shown in Figs. 4 and 5 was influenced by swap errors, we measured the distribution of response errors sorted by distractor orientation. If there were clear swap errors, then we would predict a nonuniform distribution when response errors are centered on the distractor orientation. A chi-square goodness-of-fit test for uniform distributions indicated no significant deviation from uniformity for either experiment, Experiments 3–4: χ^2^ <.005, *p* = 1.0, for all distractor–noise combinations.

## Discussion

When using memoranda and distractors that do not share a common feature, we find that stimulus noise and distractor presence and relevance exert largely additive influences on the overall magnitude of memory errors. However, using memoranda and distractors that share a common feature allowed us to more directly assess systematic interactions (see also Zhang & Lewis-Peacock, 2023). We found that distractors bias patterns of memory recall error and that these biases are generally more pronounced when memory representations are noisy and when distractors are behaviorally relevant. These results demonstrate that interactions between seen and remembered stimuli are mediated by factors that regulate the strength of internal representations such as stimulus noise, contrast, and behavioral relevance, and suggest partially overlapping mechanisms.

Importantly, the observation that ignored distractors induce small biases in Experiment 3 and a negligible bias in Experiment 4 suggests that some additional processing of distractors, beyond passive viewing, is required to observe robust sample/distractor interactions. On one account, processing a relevant distractor simply amplifies the sensory-evoked response and might lead to more interference with a sensory-like WM representation (Reynolds & Chelazzi, 2004). In contrast to this simple sensory interference model, other theoretical frameworks suggest that processing a relevant stimulus is tantamount to encoding that item into WM (Awh & Jonides, 2001; Foster et al., 2016, 2017). Accordingly, the systematic biases that we report might reflect interactions between multiple representations stored in WM as opposed to interactions between sensory representations of visible stimuli and sensory-like WM representations.

Even if behavioral biases reflect interactions between items stored in WM, it is unlikely that remembering a stimulus that is no longer present and processing a stimulus that is continuously visible results in the same depth of encoding. For example, neural markers of WM capacity such as contralateral delay activity (CDA) are selectively modulated by memory storage requirements and not by processing continuously visible stimuli (Hakim et al., 2019). In addition, neural markers of directing spatial attention to an object such as alpha-band modulations do not co-vary with other neural markers of WM load (Awh & Vogel, 2025; Fu et al., 2023; Jones et al., 2024; Oberauer, 2019). This dissociation between processing relevant stimuli and measures of WM capacity is consistent with recent work suggesting that actively monitoring a relevant stimulus leads to obligatory encoding in WM, but to varying degrees depending on task demands. For example, if a relevant stimulus is continuously visible, then there is no need to bind its identity to a particular spatiotemporal context and it is sufficient to only store item-level information about specific features in WM (e.g., the item's current orientation, color). However, remembering a stimulus that is no longer present requires binding item-level information with information about spatial and temporal context, and only this process of contextual binding is thought to contribute to WM capacity limits (Awh & Vogel, 2025; Li et al., 2025, 2026; Oberauer, 2019; Oberauer & Lewandowsky, 2019). Given this hypothesized distinction, bias induced by the visible distractor in the present experiment may reflect the influence of an item-level representation on a fully bound representation of the sample. If true, then the limited capacity mechanisms that support the storage of fully-bound WM representations may be permeable and subject to interference by less complex item-level representations, even if the item-level representations don’t significantly contribute to overall memory load.

In a parallel theoretical debate, prior work has shown similar attractive biases (Dubé et al., 2014; Hallenbeck et al., 2021; Huang & Sekuler, 2010; Lorenc et al., 2018; Mallett et al., 2020; Nemes et al., 2011, 2012; Rademaker et al., 2015; Teng & Kravitz, 2019; Van der Stigchel et al., 2007; Zhang & Lewis-Peacock, 2023), but it is unclear whether these effects are attributable to overlapping representations in early sensory brain regions or whether the distractor influences post-perceptual stages of memory storage or decision making (such as those proposed to support attractive serial dependence; e.g., Bliss et al., 2017; Papadimitriou et al., 2015; Sheehan & Serences, 2022; St John-Saaltink et al., 2016). We argue that the systematic modulations reported here are at least consistent with a partially overlapping representational format for sensory and mnemonic information as manipulations that are expected to impact the quality of low-level visual information processing, such as more stimulus noise or attentional modulations of evoked neural responses to the distractor, lead to larger interactions between representations of seen and remembered stimuli. Consistent with these interactions playing out in early visual cortex, Hallenbeck et al. (2021) showed that both behavioral reports and neural representations in early visual areas V1–V3 were drawn towards nearby distractors (± 12° from the remembered position) in a spatial working memory task. However, we cannot directly assess the cortical origins of the biases that we report here based solely on our behavioral data.

Although reformatting of memory representations is a plausible encoding strategy to help reduce interference (Libby & Buschman, 2021; Xu, 2024), there is still little direct evidence that these more abstract representations directly support memory recall. For example, Xu (2024) found that representations of remembered stimuli in early visual cortex changed as a function of distractor type, suggesting interacting sensory and mnemonic codes. In contrast, representations of remembered stimuli in parietal cortex did not vary across distractor types, consistent with untangled codes. Based on this observation, Xu proposed a privileged role for the untangled representations maintained in parietal cortex based on the logical argument that these representations should be less susceptible to interference and better able to support uncontaminated memory representations. Indeed, Xu (2024) did not observe any behavioral effects of distractor presence, a null result that is consistent with recall performance being based on untangled representations in parietal cortex. That said, Xu (2024) used a relatively small set of categorically distinct objects (e.g., coat hangers, bicycles, shoes) as stimuli, thus it is not clear what patterns of behavioral errors might arise or even whether to expect interference between such disparate stimulus classes. In contrast, we used a continuous feature space and a distractor that shared a common feature with the memory target, thus we could make precise predictions about how patterns of behavioral errors would change given interacting representations. At minimum, the amplified attractive biases we observe demonstrate that memory recall errors are not always based on representations that are recoded into abstract formats. Of course, our stimuli are more artificial compared to the naturalistic objects used in Xu (2024), and using naturalistic stimuli can impact some facets of WM storage (Brady & Störmer, 2022; Brady et al., 2016; Chung et al., 2024; Morales-Torres et al., 2024; Thibeault et al., 2024). Future work might combine the advantages of well-parameterized artificial stimulus spaces and natural images by using tools such as CNNs to make predictions about how representations of real-world objects might interact at different levels of processing to assess the role of entangled (or untangled) representations on behavioral reports.

While our results indicate that behavioral errors exhibit a bias consistent with interactions between representations of attended sensory stimuli and internal representations in WM, these behavioral data do not imply that mnemonic representations are maintained in a “pure” sensory-like format. For example, sensory representations can be recoded into compressed or transformed to more efficiently meet different task demands (e.g., collapsing a motion direction into a lower dimensional representation of orientation, or outsourcing a visual representation to motor cortex when the behavioral response is known in advance; Duan & Curtis, 2024; Henderson et al., 2022; Kwak & Curtis, 2022; Yu et al., 2020). However, the present experiments require high precision representations to support accurate recall and include feature and behaviorally relevant distractors. Therefore, relying on an abstract or lower-dimensional code is likely suboptimal and a more sensory-like representation might better support performance, even at the cost of systematic biases.

## Conclusion

The present results demonstrate that representations of remembered and processed stimuli can systematically interact, suggesting at least partial intermingling of their representations. While unlikely to be obligatory in all tasks, the interactions in the present data set are particularly prevalent when the new inputs are behaviorally relevant and the processed and remembered stimuli share a common feature. Determining the level at which these biases originate will require future work, particularly to understand how item-level WM representations might interact with capacity limited, contextually bound WM representations.

## Supplementary Information

Below is the link to the electronic supplementary material.

Supplementary file1 (DOCX 2754 kb)

## Data Availability

All materials, data, and analysis scripts are publicly available online (https://osf.io/xdte2/?view_only=32273d55e2c04ad787442758d0941c61). No aspects of Experiments 1 and 2 were preregistered. For Experiments 3 and 4, the design, sample size, exclusion criteria, and analysis plans were preregistered on OSF using the AsPredicted template (Experiment 3: https://osf.io/k76dh; Experiment 4: https://osf.io/eafkd).
