# Supplementary material for: Memory recall errors reflect interacting sensory and mnemonic representations
Source: Psychon Bull Rev. 2026 Aug 10;33(6):206. doi: 10.3758/s13423-026-02979-4 (PMC13457412; doi:10.3758/s13423-026-02979-4)
Supplement: Supplementary file 1 [file 13423_2026_2979_MOESM1_ESM.docx]

**Supplementary Material**

I. **Bayesian ANOVA**

Calculated with BayesFactor package in R (version 0.9.12.4.7).

A. *Experiment 1:* There was a main effect of stimulus noise such that errors increased with stimulus noise (BF_10_ = 4.63 x 10^15^). There was a main effect of distractor condition such that errors increased with the presence of distractors (BF_10_ = 27.13). There was evidence that supports the null hypothesis that there was no interaction between stimulus noise and distractors (BF_10_ = 0.05).
B. *Experiment 2:* There was a main effect of stimulus noise such that errors increased with stimulus noise (BF_10_ = 1.54 x 10^34^). There was a main effect of distractor condition such that errors increased with the presence of distractors (BF_10_ = 4.87). There was evidence that supports the null hypothesis that there was no interaction between stimulus noise and distractors (BF_10_ = 0.02).
C. *Experiment 3 (pre-registered):* There was a main effect of stimulus noise such that errors increased with stimulus noise (BF_10_ = 8.87 x 10^14^). There was a main effect of distractor condition such that errors increased with the presence of distractors (BF_10_ = 1.79 x10^4^). There was evidence that supports the null hypothesis that there was no interaction between stimulus noise and distractors (BF_10_ = 0.16).
D. *Experiment 4 (pre-registered):* There was a main effect of stimulus noise such that errors increased with stimulus noise (BF_10_ = 1.67 x 10^5^). There was minimal evidence that attending to distractors increased errors (BF_10_ = 1.10). Evidence of an interaction was inconclusive (greater than 1/3) (BF_10_ = 0.35).

E. Pairwise comparisons

**Table S1.** Experiment 2:

| Stimulus Noise | Distractor Condition | BF_10_ |
| --- | --- | --- |
| Low | None vs. Ignore | 0.37 |
|  | None vs. Process | 0.63 |
|  | Ignore vs. Process | 0.18 |
| Medium | None vs. Ignore | 7.94 |
|  | None vs. Process | 4.16 |
|  | Ignore vs. Process | 0.21 |
| High | None vs. Ignore | 0.88 |
|  | None vs. Process | 3.13 |
|  | Ignore vs. Process | 0.20 |

**Table S2.** Experiment 3:

| Stimulus Noise | Distractor Condition | BF_10_ |
| --- | --- | --- |
| Low | None vs. Ignore | 3.88 x 10^2^ |
|  | None vs. Process | 2.54 x 10^4^ |
|  | Ignore vs. Process | 8.61 |
| High | None vs. Ignore | 87.8 |
|  | None vs. Process | 5.90 x 10^2^ |
|  | Ignore vs. Process | 0.48 |

II. **Pairwise Comparisons - Linear Mixed Effects Model**

For pairwise comparisons we used the emmeans package (version 2.0.2). P-values are adjusted with Holm correction for multiple comparisons.

**Table S3.** Experiment 2:

| Stimulus Noise | Distractor Condition | Estimate | SE | z | p_Holm_ |
| --- | --- | --- | --- | --- | --- |
| Low | None vs. Ignore | -0.056 | 0.039 | -1.44 | 0.30 |
|  | None vs. Process | -0.071 | 0.039 | -1.82 | 0.21 |
|  | Ignore vs. Process | -0.015 | 0.044 | -0.34 | 0.73 |
| Medium | None vs. Ignore | -0.112 | 0.039 | -2.88 | < 0.01 |
|  | None vs. Process | -0.100 | 0.039 | -2.58 | < 0.05 |
|  | Ignore vs. Process | 0.012 | 0.044 | 0.27 | 0.79 |
| High | None vs. Ignore | -0.075 | 0.039 | -1.92 | 0.11 |
|  | None vs. Process | -0.151 | 0.039 | -3.88 | < 0.01 |
|  | Ignore vs. Process | -0.076 | 0.044 | -1.73 | 0.11 |

**Table S4**. Experiment 3:

| Stimulus Noise | Distractor Condition | Estimate | SE | z | p_Holm_ |
| --- | --- | --- | --- | --- | --- |
| Low | None vs. Ignore | -0.095 | 0.030 | -3.15 | < 0.01 |
|  | None vs. Process | -0.210 | 0.030 | -7.00 | < 0.01 |
|  | Ignore vs. Process | -0.116 | 0.030 | -3.84 | < 0.01 |
| High | None vs. Ignore | -0.102 | 0.030 | -3.37 | < 0.01 |
|  | None vs. Process | -0.182 | 0.030 | -6.05 | < 0.01 |
|  | Ignore vs. Process | -0.080 | 0.030 | -2.67 | < 0.01 |

III. **Experiment 4 sample size 12.** Experiment 4 was pre-registered with the goal sample size of 12 participants. After pre-registration, an updated sample size was estimated using Experiment 3’s data, Experiment 4 data collection continued for the additional 5 participants needed to reach the new goal sample size. The following are the same analyses reported in the main text with the original smaller sample size.
     A. *Mean memory error*: Working memory response errors were quantified using absolute error and summarized using the linear mixed effect model described in the main text. There was a significant main effect of stimulus noise (*F*(1,11) = 9.65, *p* < .01, 𝜔_p_^2^ = 0.40). There was a significant main effect of distractor condition (*F*(1,11) = 12.8, *p* < .01, 𝜔_p_^2^ = 0.47). There was no significant interaction between stimulus noise and distractor (*F*(1, 18974) = 2.07, *p* = 0.15, 𝜔_p_^2^ = 5.66 x 10^-5^).
     B. *Memory Bias*: Working memory response error bias was quantified using the amplitude of the error distributions binned by distractor-sample (Δθ) as described in the main text. Attending to a distractor produced memory errors that were attracted to the distractor orientation (permutation test 95% CI [1.28, 1.95], p < 0.001). There is no significant effect of stimulus noise on bias (Experiment 4a: permutation test 95% CI [-0.22, 0.56], *p* = 0.40) (Figure S5).

**S5.** Experiment 4, sample size 12.

**S6.** Experiment 1+2 combined.

**S7.** Experiment 3+4 combined.

IV. **Sensitivity analysis for stimulus noise and distractor Interaction**

We conducted sensitivity analyses (*simr 1.0.7,* *nsim* = 1000, 𝛼 = 0.05) for all experiments. We used a simplified linear mixed model with random intercepts for participants only, as the fuller random effects structure used in the main analyses (which additionally included by-participant random intercepts for target and distractor condition) produced singular fits with the sample sizes required for simulation. The simulations suggest that we had enough data to detect the following interaction effects at 80% power (E1: β = 0.025 or a 2.57% change in absolute error; E2: β = 0.029 or a 2.89% change in absolute error; E3: β = 0.025 or a 2.52% change in absolute error; E4: β = 0.023 or a 2.36% change in absolute error).

V. **Relaxed exclusion criteria**.

The exclusion criteria were pre-registered to allow no more than 10% no-response trials, where the subject failed to make any button press within the allotted response time during the trial. The following are repeated analyses where possible using a more relaxed exclusion criterion of 20%.

A. *Experiment 1 Mean memory error*: Relaxing the exclusion criteria from 10% to 20% no-response trials per subject allowed 4 out of 8 previously excluded subjects to be added to the sample (n = 38 total). There was a significant main effect of stimulus noise (*F*(2, 72.1) = 28.1, *p* < .001, 𝜔_p_^2^ = 0.42). There was no significant main effect of distractor condition (*F*(2, 75.0) = 2.97, *p* = 0.06, 𝜔_p_^2^ = 0.05). There was no significant interaction between stimulus noise and distractor condition (*F*(4, 12938.2) = 0.99, *p* = 0.41, 𝜔_p_^2^ = 0.00).
B. *Experiment 2 Mean memory error*: Relaxing the exclusion criteria from 10% to 20% no-response trials per subject allowed 4 out of 8 previously excluded subjects to be added to the sample (n = 39 total). There was a significant main effect of stimulus noise (*F*(2, 79.9) = 52.4, *p* < .001, 𝜔_p_^2^ = 0.55). There was a significant main effect of distractor condition (*F*(2, 79.8) = 10.8, *p* < .001, 𝜔_p_^2^ = 0.19). There was no significant interaction between stimulus noise and distractor condition (*F*(4, 13374.0) = 0.86, *p* = 0.49, 𝜔_p_^2^ = 0.00).
C. *Experiment 3* Mean memory error: Relaxing the exclusion criteria from 10% to 20% no-response trials per subject allowed 3 out of 10 previously excluded subjects to be added to the sample (n = 27 total). There was a significant main effect of stimulus noise (*F*(1, 26.0) = 31.1, *p* < .001, 𝜔_p_^2^ = 0.52). There was a significant main effect of distractor condition (*F*(2, 51.9) = 39.7, *p* < .001, 𝜔_p_^2^ = 0.58). There was no significant interaction between stimulus noise and distractor condition (*F*(2, 15196.8) = 0.01, *p* = 0.99, 𝜔_p_^2^ = 0.00).
D. *Experiment 3* Bias: There was a significant main effect of stimulus noise (*permutation test 95% CI [0.31, 0.63], p < 0.001*). There was a significant main effect of distractor condition (*permutation test 95% CI [2.18, 2.62], p < 0.001*). There was a significant interaction between stimulus noise and distractor condition (*permutation test 95% CI [0.74, 1.67], p < 0.01*).
E. *Experiment 4 Mean memory error*: Relaxing the exclusion criteria from 10% to 20% no-response trials per subject allowed 3 out of 7 previously excluded subjects to be added to the sample (n = 20 total). There was a significant main effect of stimulus noise (*F*(1, 19) = 15.5, *p* < .001, 𝜔_p_^2^ = 0.41). There was a significant main effect of distractor condition (*F*(1, 19) = 39.7, *p* < .01, 𝜔_p_^2^ = 0.36). There was a significant interaction between stimulus noise and distractor condition, but the effect size was negligible (*F*(1, 31544) = 7.42, *p* < .01, 𝜔_p_^2^ = 2.03 x10^-4^).
F. *Experiment 4* Bias: There was not a significant main effect of stimulus noise (*permutation test 95% CI [-0.05, 0.79], p = 0.20*). There was a significant main effect of distractor condition (*permutation test 95% CI [1.04, 1.68], p < .001*). There was no significant interaction between stimulus noise and distractor condition (*permutation test 95% CI [-0.73, 0.40], p = 0.46*).
